# Independent and Additive Effects of Glutamic Acid and Methionine on Yeast Longevity

**DOI:** 10.1371/journal.pone.0079319

**Published:** 2013-11-07

**Authors:** Ziyun Wu, Lixia Song, Shao Quan Liu, Dejian Huang

**Affiliations:** 1 Food Science and Technology Programme, Department of Chemistry, National University of Singapore, Singapore, Republic of Singapore; 2 National University of Singapore (Suzhou) Research Institute, Suzhou, Jiangsu, People's Republic of China; University College London, United Kingdom

## Abstract

It is established that glucose restriction extends yeast chronological and replicative lifespan, but little is known about the influence of amino acids on yeast lifespan, although some amino acids were reported to delay aging in rodents. Here we show that amino acid composition greatly alters yeast chronological lifespan. We found that non-essential amino acids (to yeast) methionine and glutamic acid had the most significant impact on yeast chronological lifespan extension, restriction of methionine and/or increase of glutamic acid led to longevity that was not the result of low acetic acid production and acidification in aging media. Remarkably, low methionine, high glutamic acid and glucose restriction additively and independently extended yeast lifespan, which could not be further extended by buffering the medium (pH 6.0). Our preliminary findings using yeasts with gene deletion demonstrate that glutamic acid addition, methionine and glucose restriction prompt yeast longevity through distinct mechanisms. This study may help to fill a gap in yeast model for the fast developing view that nutrient balance is a critical factor to extend lifespan.

## Introduction

Reduction of food intake by 10–40% extends lifespan in diverse animals, including spiders, beetles, fish, and dogs, as well as the commonly used laboratory-model organisms: yeast, worms, fruit flies, and mice. Calorie restriction (CR) is frequently reported as the most robust non-genetic intervention to extend lifespan and health span. In 2009, the results of a 20-year caloric restriction study in rhesus monkeys at the Wisconsin National Primate Research Center (WNPRC) suggested that CR might ameliorate human aging, because the monkeys with 70% of *ad libitum* food supply had fewer age-related deaths and lower incidence of diabetes, cancer, cardiovascular disease, and brain atrophy [1]. However, a recent publication reported that diet composition might significantly affect the longevity of CR in rhesus monkeys at the National Institute on Aging (NIA). In the later study, CR reduced the incidence of diabetes and cancer but did not lower rates of cardiovascular disease and age-related death [2]. A notable difference between the two studies is diet composition. The NIA study diet had relatively diverse and balanced nutrients, whereas the WNPRC study used purified components with high sucrose content [2].

In addition to the two studies in primates, the idea that balance of nutrients in the diet might be a better way than simple dietary restriction for healthy lifespan was reported in other organisms [3], [4], [5], [6], [7]. However, this emerging idea is not well substantiated in yeast. The benefits of dietary restriction have been generally suggested to arise from intake of fewer calories (termed caloric restriction). Thus, influences of nutrients on the biological aging process might be more dependent on the macronutrients including carbohydrates, fats and proteins [8]. In addition to carbohydrates and fats, proteins (amino acids) in the diet as another energy contributor was shown to mediate lifespan significantly in commonly used aging model organisms, namely yeast [9], [10], fruit flies [5], [6], and mice [11], [12]. Notably, methionine may be a special one among various amino acids to regulate lifespan, since restriction of methionine by 80% was reported to increase median and maximal lifespan by 30% and 40% in rats [13].

The budding yeast (*Saccharomyces cerevisiae*) serves as a leading model organism for studying evolutionarily conserved mechanisms relevant to human aging and age-related diseases [14], [15], [16], [17], [18]. There are two aging models in the budding yeast: replicative aging and chronological aging [15]. Although both types of yeast aging are influenced by nutrient composition of media, the relationship between nutrition and lifespan is unclear. It is widely known that moderate glucose restriction slows yeast chronological and replicative aging significantly. Our recent study revealed that the chronological lifespan extension by a typical glucose restriction regime was dependent on the nutrients in media and that medium composition was a key determinant for yeast longevity. The three nutrients (glucose, total amino acids and YNB) and their interactions played important roles in affecting lifespans of different yeast strains [19]. Modification of the composition of amino acids in a medium has been also reported to change yeast lifespan [10], [20], [21], [22]. Yet, comprehensive studies are still lacking with regard to evaluate the influence of individual amino acids on yeast chronological lifespan in standard synthetic defined (SD) medium condition. Reported here is our finding.

## Materials and Methods

### Materials

The wild-type strain *S. cerevisiae* BY4742 (MATα *his3*Δ1 *leu2*Δ0 *lys2*Δ0 *ura3*Δ0) and single gene deletion mutant strains in the BY4742 genetic background were obtained from Thermo Scientific Open Biosystems (Huntsville, AL, USA). The culture of each yeast reference strain was aliquoted into 10 μL and stored at –80°C. L-amino acids were from GL Biochem (Shanghai, China), yeast nitrogen base w/o amino acids and ammonium sulfate (YNB), peptone, agar, yeast extract were from Amresco (Solon, OH, USA). YPD Broth and other chemicals were from Sigma-Aldrich Chemical Company (St Louis, MO, USA). HPLC grade acetonitrile and methanol were obtained from Tedia Company (Fairfield, OH, USA). The 96-well polystyrene microplates with flat bottom were purchased from Fisher Scientific (Nunc, Rochester, NY, USA). Other solvents were of HPLC grade from commercial sources.

### Lifespan assay

The determination of chronological lifespan (CLS) of yeast was carried out according to the method described previously [23], [24]. In brief, the yeast cells were prepared by transferring a streaked strain from frozen stocks onto YPD (1% yeast extract/2% peptone/2% dextrose) agar plates. After incubating the cells at 30°C for 2 days or until colonies appeared, a single colony was picked and inoculated into 1.0 mL YPD liquid medium in a 4-mL glass vial and cultured at 30°C for 2 days in a flat incubator at 200 rpm. The 2-day YPD culture was washed with autoclaved 18 MΩ.cm milli-Q grade water twice and diluted with water (1:10), then stored in refrigerator at 4°C for at least 24 h. After incubation at 4°C for one day, 5 µL (≈ 1×10^4^ cells) of the diluted culture was transferred to 1.0 mL of synthetic defined (SD) media (**Table 1**) supplying with different nutrients composition and maintained at 30°C, 200 rpm for the chronological aging experiment. For growth in buffered medium, a citrate phosphate buffer (64.2 mM Na_2_HPO_4_ and 17.9 mM citric acid, pH 6.0) adjusted to pH 6.0 was added to the medium prior to inoculation. After 2 days of culture in aging media, the cells reached stationary phase and the first age-point was ready to be taken. Subsequent age-points were taken every 2–4 days. For each age-point, 5.0 µL of the mixed culture was pipetted into each well of 96-well microplate. One hundred microliter YPD medium was then added to each well. The cell population was monitored with a PowerWave XS microplate reader (BioTek, Winooski, VT, USA) by recording OD660 every 5 min during 12–24 h.

**Table 1:** Composition of synthetic defined (SD) medium used for yeast CLS analysis.

Component	Concentration (1×)
**Glucose**	20 g/L
**Yeast Nitrogen Base** without amino acid and ammonium sulphate	1.7 g/L
**Ammonium sulphate**	5 g/L
**Amino acids (1×)**	
Essential	
*Uracil*	100 mg/L
*L-histidine*	100 mg/L
*L-leucine*	300 mg/L
*L-lysine-HC1*	150 mg/L
Non-Essential	
*Adenine*	80 mg/L
*L-arginine*	40 mg/L
*L-aspartic acid*	100 mg/L
*L-glutamic acid*	100 mg/L
*L-methionine*	80 mg/L
*L-phenylalanine*	50 mg/L
*L-serine*	400 mg/L
*L-threonine*	200 mg/L
*L-tryptophan*	200 mg/L
*L-tyrosine*	40 mg/L
*L-valine*	150 mg/L
*L-isoleucine*	60 mg/L

### Biomass production assay

Biomass of each aging vial at one age-point was measured as the average reading of OD values at 660 nm from 10 to 30 min in outgrowth curves, and the total biomass production of each medium was defined as the mean biomass from day 6 to day 10.

### Yeast cell growth assay

After one day incubation of the diluted culture at 4°C, the cells were washed twice with water to remove other nutrients, 5.0 µL (≈ 1×10^4^ cells) of the diluted cells was pipetted into each well of 96-well microplate. One hundred µL of different media was then added to each well. The cell population was monitored with a microplate reader by recording the OD every 5 min at 660 nm.

### Acetic acid and pH analysis

The aging culture (2 day) was centrifuged at 4,000 g for 10 min at room temperature. The supernatant was collected and stored at –20°C before analysis of acetic acid and pH. The pH of supernatant was measured using a Eutech Ion 6+ pH meter with a micro-tip pH electrode (Eutech Instruments, Singapore). The supernatant was filtered through a Sartorius Minisart polytetrafluoroethylene (PTFE) membrane (0.2 µm) before HPLC analysis. The acetic acid analysis was performed on a Waters HPLC system (Milford, MA, USA) with an Alliance 2659 separation module, a 2996 photodiode array (PDA) detector. The detection wavelength was set at 210 nm. The column used was a Supelcogel C-610H column (300×7.8 mm, Supelco) with 0.1% sulphuric acid as mobile phase. Each sample was run 60 min at a flow rate of 0.4 mL/min at room temperature.

### Data analysis

The raw data from the microplate reader were exported to Excel (Microsoft, San Leandro, CA, USA). From the growth curves, the viability of the yeast can be obtained according to our previous report [24]. Survival integral (SI) of each aging culture is defined as the area under the survival curves (AUC). The viability of the yeast was obtained according to our previous report [24]. The analysis of variance for each set of biological replicates was carried out with the SAS statistical program, and differences between the means of SI for treatments were determined by Duncan’s multiple range test at *P*<0.05 or *P*<0.01.

## Results

### Ratio of essential and non-essential amino acids regulates lifespan and biomass changes

There are usually 14 amino acids and two bases, adenine and uracil, in a standard synthetic defined (SD) medium (**Table 1**), only histidine, leucine, lysine and uracil are essential for wild-type yeast strain BY4742 (MATα *his3*Δ1 *leu2*Δ0 *lys2*Δ0 *ura3*Δ0) [25]. We first examined whether ratio of essential amino acids (EAA) to non-essential amino acids (NEAA) caused chronological lifespan (CLS) alteration. The EAA and NEAA contents from 0.2-fold to 5-fold of normal conditions were tested.

**Figure 1:**
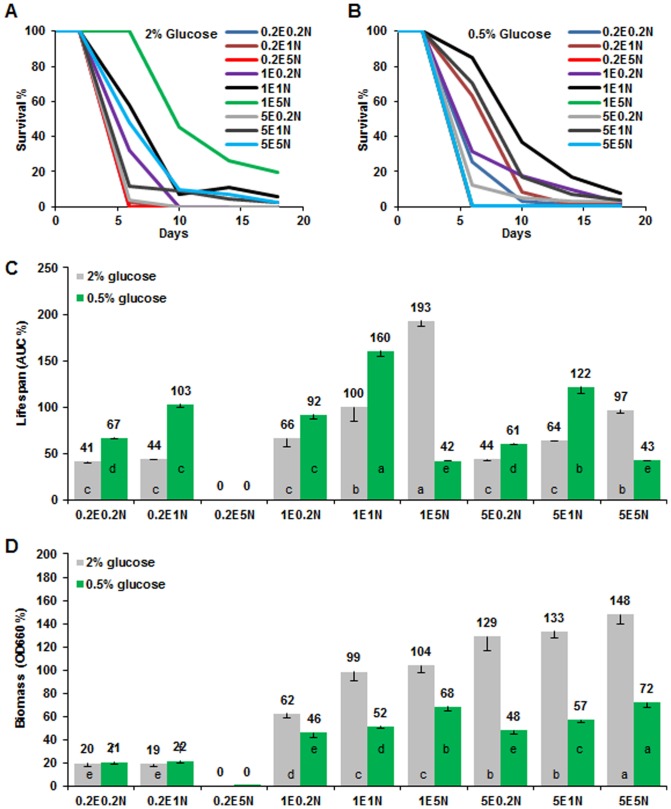
Ratio of essential and non-essential amino acids regulates lifespan and biomass changes. Survival curves of wild type yeast (BY4742) were cultured in SD media with different ratios of EAA (0.2 to 5 fold) and NEAA (0.2 to 5 fold) in normal (2% glucose, **A**) and glucose restriction (0.5% glucose, **B**) conditions. The normalized lifespan and biomass comparison were presented in **C** and **D**, respectively. All tests were based on SD media. 1E5N represents SD medium containing 1-fold EAA and 5-fold NEAA. The EAA and NEAA compositions and concentrations (1 fold) were listed in **Table 1**. Area under the survival curve (AUC) represents the survival integral for lifespan comparison and AUC of the control (1E1N with 2% glucose) was defined as 100%. Biomass production was measured as the average values at OD660 of each medium from day 6 to day 10 and biomass of the standard SD medium (1E1N with 2% glucose) was defined as 100%. Lifespan and biomass: mean ± s.e.m (n = 4); compared using Duncan’s multiple range test at *P*<0.01 and different lowercase letters in columns indicate significant difference.

**Figure 2:**
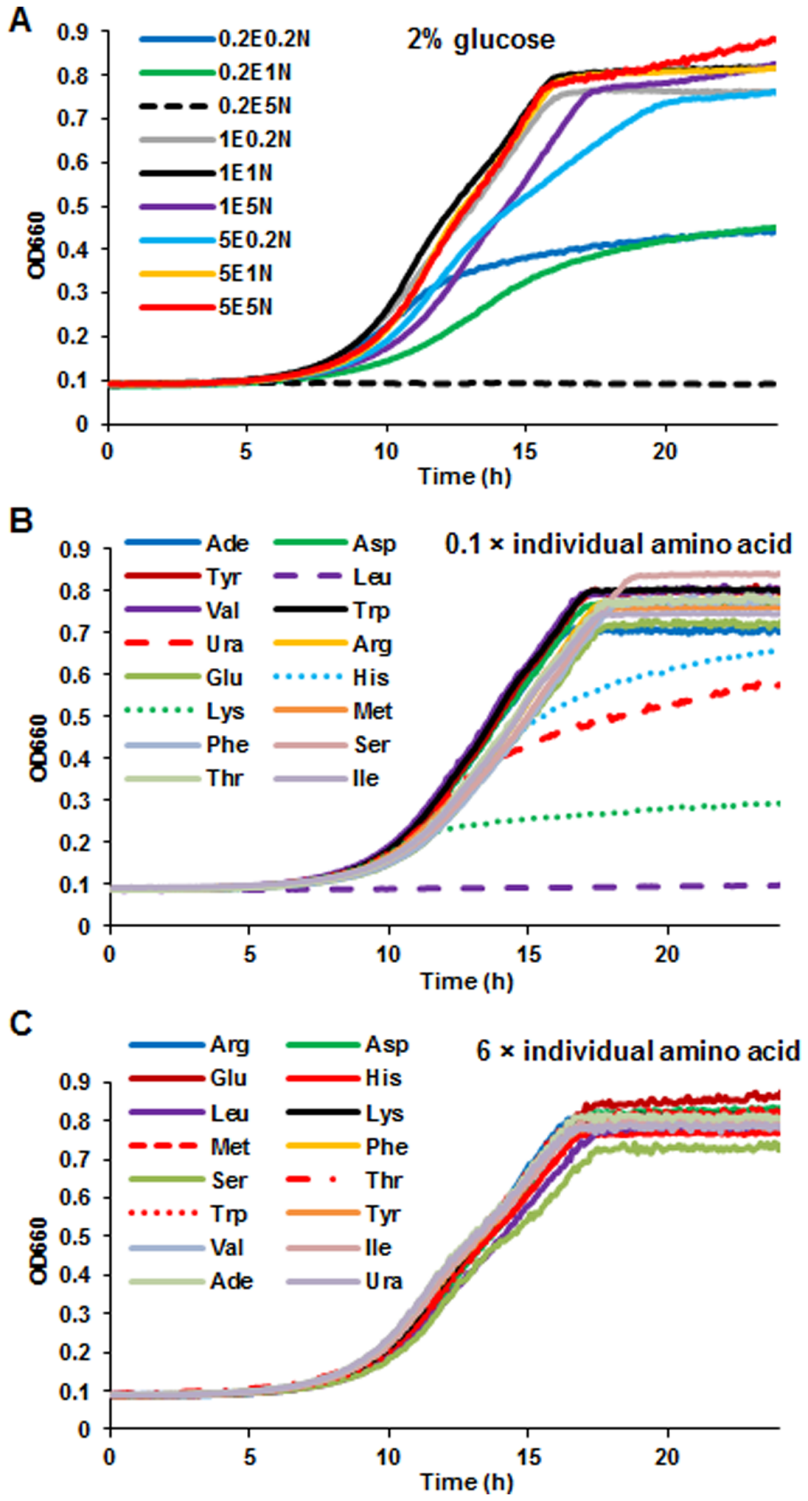
Growth curves of wild type yeast cultured in media with diverse amino acids compositions. (**A**) Ratio of EAA and NEAA was able to prevent yeast proliferation. (**B**) Reduction of yeast EAAs (leucine, lysine, uracil, histidine) inhibited cells growth and biomass production. (**C**) Increase of individual amino acids does not affect yeast growth. Wild type yeast (BY4742) (≈ 1×10^4^ cells) cells were grown in each well of 96-well microplate containing 100 µL of different media. The cell population was monitored with a microplate reader by recording the OD every 5 min at 660 nm. All tests were based on SD media. 1E5N represents SD medium containing 1-fold EAA and 5-fold NEAA.

The ratio of EAA and NEAA changed lifespan substantially. The composition of 1-fold EAA and 5-fold NEAA had an optimal lifespan under normal glucose condition (2%, **Figure 1A, C**). However, this composition produced significantly shorter CLS under calorie restriction (CR) condition (0.5% glucose), which gave an optimal lifespan under standard composition (1E1N, 1-fold EAA and 1-fold NEAA) (**Figure 1B, C**). These results suggest that the EAA and NEAA mediated lifespan alteration is influenced by glucose content in the medium (**Figure 1C**), and this is consistent with previous finding that total amino acids content has close interaction with glucose to regulate yeast CLS [19] and that the ratio of protein and carbohydrate was able to significantly change lifespan in *Drosophila melanogaster* [3], [8]. Furthermore, our data suggest that high NEAA could suppress the CR effect on CLS, since CR extended CLS under conditions of 0.2 and 1 fold NEAA with 0.2 to 5 fold EAA, while CR did not appear to extend CLS in the presence of 5-fold NEAA in comparison with that under the 2% glucose media (**Figure 1C**). Very different trend was observed for the cell biomass, which showed that high EAA and NEAA (5E5N) promoted high cell biomass production (**Figure 1D**). This observation is similar to literature reports that lifespan, but not biomass/reproduction, is optimized by CR in yeast and other high organisms [18], [24], [26], [27]. It is remarkable that, 0.2-fold EAA and 5-fold NEAA totally inhibited cell growth (**Figure 2A**), while the other ratios produced different amounts of cell biomass but did not interrupt cell proliferation. Although we do not know the mechanisms, this result illustrated that amino acid composition was important for cell growth. It is reported that removal of non-essential amino acid serine could inhibit proliferation of p53-deficient cancer cells [28]. In our case, excessive NEAA may suppress the uptake of EAA in 0.2E5N media.

**Figure 3:**
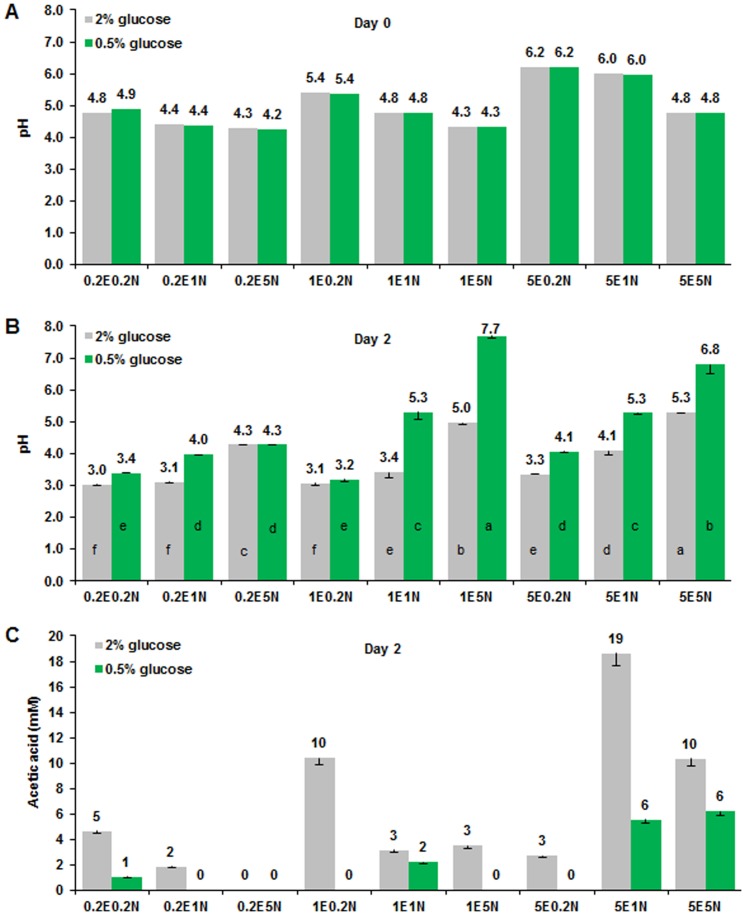
Effects of EAA and NEAA on pH values and acetic acid production of aging media. (**A**) EAA and NEAA composition slightly altered the pH of media. (**B**) The pH of aging media at stationary phase (day 2) was dependent on glucose, EAA and NEAA in media. Differences in means of the pH values of different media under normal or CR conditions, respectively, were analyzed by Duncan’s multiple range test at *P*<0.01. (**C**) Acetic acid concentrations in aging media (day 2) was changed by modifications of glucose, EAA and NEAA in media. Datum 0 means not detectable. The pH values of the different fresh media (day 0) were measured once. Acetic acid and pH values (day 2) were expressed as mean ± s.d. (n = 3).

The pH and acetic acid in aging media were reported to influence yeast chronological aging [29], [30]. We measured the pH (**Figure 3A, B**) and acetic acid concentration (**Figure 3C**) of the stationary phase culture at day 2 but found that the pH was slightly affected by the ratio of EAA and NEAA. High EAA increased pH value of the medium (day 0); in contrast, high NEAA reduced the pH value (**Figure 3A**). After 2 day of culture, the pH of medium had substantial changes (**Figure 3B**). For example, 1E5N had the highest pH value under CR conditions, but it did not produce longer CLS (**Figure 1C**). In most cases, CR induced higher pH value, longer CLS, lower biomass production, and lesser acetic acid accumulation than that under the normal glucose conditions. However, acidification and acetic acid of aging medium had a low correlation with lifespan and biomass (correlation coefficient are very low, data not shown), and the medium with lower acidification did not always result in longer lifespan, which could be due to the influence of amino acid composition in the SD medium.

### Methionine and glutamic acid cause lifespan and biomass alterations

**Figure 4:**
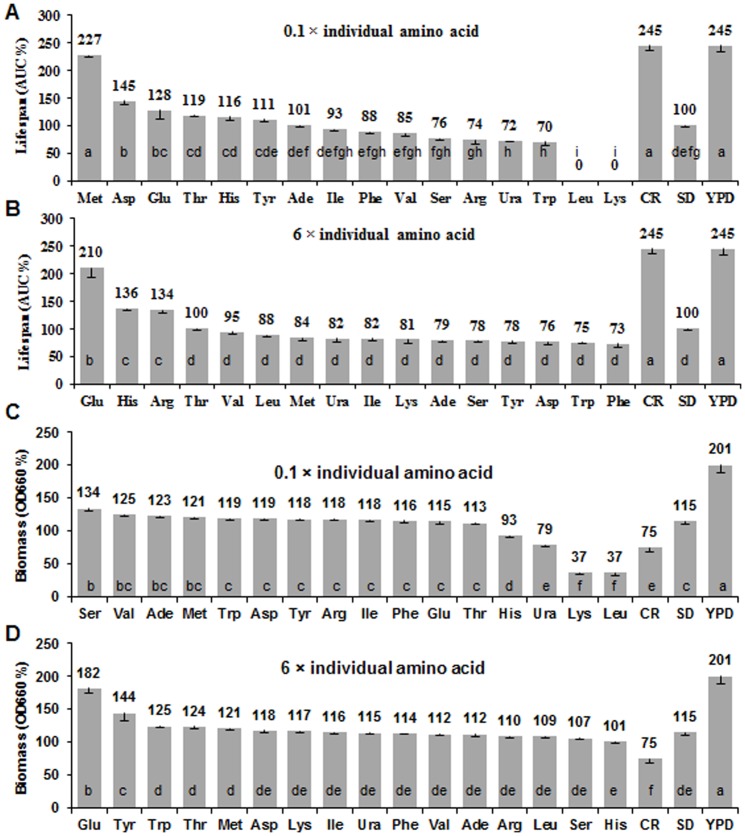
Effect of individual amino acids on yeast lifespan and biomass production. Lifespan comparison of wild type yeast was grown in SD media with individual amino acid restriction (**A**) or addition (**B**). The biomass productions were shown in **C** and **D**, respectively. To reduce (0.1-fold) or increase (6-fold) single amino acids in SD medium, the other amino acids concentrations were held constant. SD is standard medium with 2% glucose as the control condition in this study (**Table 1**); CR is SD medium with 0.5% glucose as the calorie restriction condition; YPD is a commonly used nutrient-rich medium with 1% yeast extract, 2% peptone and 2% dextrose. The concentrations (1 fold) of individual amino acids were listed in **Table 1**. AUC represents the survival integral for CLS comparison and AUC of the SD medium was defined as 100%. Biomass of the SD medium was defined as 100%. Data are expressed as mean ± s.e.m. (n = 6); comparison were made using Duncan’s multiple range test at *P*<0.05 and different lowercase letters in columns indicate significant differences.

We next determined whether reduction or supplementation of individual amino acids was able to change yeast CLS. To our surprise, methionine restriction (reduced to 10%, or at 8 mg/L) was sufficient and the most effective in extending lifespan to 2.3 times compared to standard SD medium. Restriction of aspartic acid also has significant lifespan extension effect but much less effective than methionine restriction (**Figure 4A**). It is noteworthy that the reduction of leucine and lysine caused low biomass production and fast loss of cell viability (**Figure 4A, C**). Conversely, an increase of non-essential glutamic acid (6-fold, or at 600 mg/L) prolonged yeast lifespan to 2.1 times relative to standard SD medium (**Figure 4B**). In addition biomass production was increased to 1.6 times of that under standard SD condition. The other essential or non-essential amino acids had not much significant effects on lifespan and biomass (**Figure 4B, D**). Although, as one would expect, restriction of the four individual EAA impaired biomass production and inhibited cell growth (**Figure 2B**), an increase of individual AA or decrease of individual NEAA could not alter biomass (**Figure 4**) and cell proliferation (**Figure 2B, C**). Overall, methionine and glutamic acid were showed to be more efficient than the other amino acids to extend yeast CLS and thus warrant more investigation.

**Figure 5:**
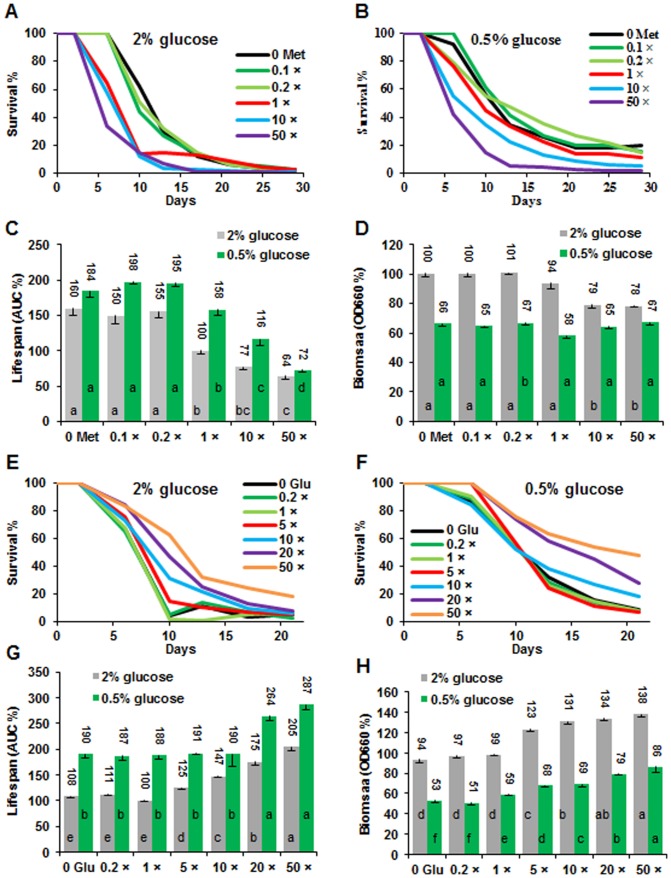
Methionine and glutamic acid cause lifespan and biomass alterations in yeast. Survival curves of wild-type yeast were cultured in normal (2% glucose, **A**) and CR (0.5% glucose, **B**) media containing different methionine levels (0 to 50×, 0 to 4000 mg/L). CLS (**C**) and biomass (**D**) comparisons of yeast were incubated in media with diverse methionine levels. AUC or biomass of the control (1× Met with 2% glucose) was defined as 100%. Survival curves of wild-type yeast grown in normal (**E**) and CR (**F**) conditions containing different glutamic acid levels (0 to 50×, 0 to 5000 mg/L). CLS (**G**) and biomass (**H**) comparisons of yeast were grown in media with diverse glutamic acid levels. AUC or biomass of the control (1× Glu with 2% glucose) was defined as 100%. Data were expressed as mean ± s.e.m. (n = 4) (**C** and **D**), n = 6 (**G** and **H**); compared using Duncan’s multiple range test at *P*<0.05 and different lowercase letters in columns indicate significant difference.

We subsequently determined the dose-response relationship of methionine and glutamic acid in lifespan extension capacity. Depletion (0) or restriction (0.1× or 0.2×) of methionine extended CLS, whereas high doses shortened CLS under normal (2% glucose) and CR (0.5% glucose) conditions (**Figure 5A-C**). Methionine restriction had little effect on yeast biomass (**Figure 5D**), cell growth (**Figure 2B**, **S1**), pH, and acetic acid of aging media (**Figure S2**). However, high doses of methionine lessened biomass and slightly enhanced acetic acid production of aging culture under normal conditions.

In contrast, addition of glutamic acid prolongs CLS and biomass production in a clear dose-response manner (from 5× to 50×) under 2% glucose media but the trend is less apparent under 0.5% glucose media. On the other hand, restriction of glutamic acid did not shorten CLS (**Figure 5G**) or reduce the biomass production and high glutamic acid concentrations did not interrupt cell growth (**Figure 2C**, **S1**). Although glutamic acid addition impaired acetic acid production, the pH of different media at day 2 fall in a small range of 3.8 to 3.5 (**Figure S2**). As an acidic amino acid, increasing the glutamic acid concentration contributes to the reduction of the initial pH values of the media from 4.7 (1×) to 3.4 (50×). In addition, the added glutamic acid lowered the acetic acid production (**Figure S2**). It is known that high dose of acetic acid is toxic to yeast cells and shorten lifespan [29]. It is plausible that the reduced production of acetic acid at increased amount of glutamic acid might be the reason for its lifespan extension effect. However, the higher acetic acid level in glutamic acid restricted media did not result in shorter lifespan. Overall, the lack of correlation we observed between acetic acid (or pH) with lifespan (**Figure 5**, **S2**) suggests that acetic acid and acidification might not be the determining factor in mediating yeast longevity caused by methionine restriction or glutamic acid addition.

### Independent and additive effects of glutamic acid, methionine and glucose on lifespan extension

**Figure 6:**
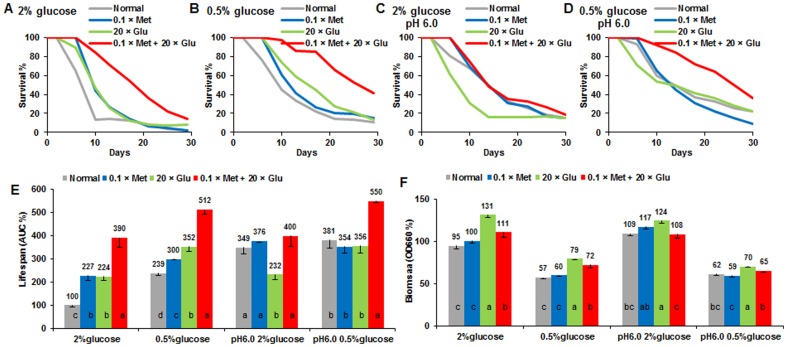
Independent and additive effects of glutamic acid, methionine and glucose on yeast lifespan extension. Survival curves of wild-type yeast were cultured in un-buffered normal (2% glucose, **A**) and CR (0.5% glucose, **B**) conditions or pH (6.0) buffered normal (**C**) and CR (**D**) conditions without (Normal/1E1N, control) or with low methionine (0.1× Met, 8 mg/L), high glutamic acid (20× Glu, 2 g/L) or a combination of both (0.1× Met + 20× Glu). CLS (**E**) and biomass (**F**) comparison of wild type yeast were grown in different media. The pH controlling was accomplished by citrate phosphate buffer solution (64.2 mM Na_2_HPO_4_ and 17.9 mM citric acid, pH 6.0). AUC represents the survival integral for lifespan comparison. AUC or biomass of the un-buffered SD medium (1E1N with 2% glucose) was defined as 100%. Data were expressed as mean ± s.e.m. (n = 6) and compared using Duncan’s multiple range test at *P*<0.05. Different lowercase letters in columns indicate significant difference.

We suspect that longevity effects of methionine restriction and glutamic acid addition might have additive effects on yeast lifespan. Hence we tested yeast grown in normal and CR conditions with 0.1-fold methionine (0.1× Met, 8 mg/L), 20-fold glutamic acid (20× Glu, 2 g/L) or a combination of both (0.1× Met + 20× Glu referred to as “combination treatment” thereto). We found that yeast CLS was greatly extended by the combination in 2% glucose conditions and could be further enhanced by CR (**Figure 6A, B**). The trend of additive effect is obvious for glutamic acid addition, and methionine restriction, and CR on yeast longevity extension.

We also examined the CLS of yeast cultured in media with 1-fold EAA and 5-fold NEAA (1E5N), since this composition increases CLS and thus possibly has additive effects with glutamic acid, methionine and CR on yeast longevity. Under this composition, methionine restriction or glutamic acid addition could still extend CLS under normal and CR conditions, but the combination treatment could not further promote the longevity in normal (2% glucose), CR (0.5% glucose) and pH buffered conditions (**Figure S3A-D**). In addition, the composition 1E5N caused significant lifespan reduction in CR conditions (**Figure S3B**) and the combination treatment had longer CLS extension in 1E1N than in 1E5N (**Figure S4**). Taken together, methionine restriction and glutamic acid addition had no additive effect on longevity extension under 1E5N composition. However, it was still sufficient to extend lifespan independent of CR, 1E5N and pH neutralization (**Figure S4**).

It was known that buffering the pH of the aging medium or adjusting the pH to 6.0 could greatly protect against yeast lifespan reduction [29]. Acidification of aging medium was proposed as a major factor to accelerate yeast aging [15], [29], [30], [31], [32]. We thus examined CLS extension effects of methionine restriction, glutamic acid addition, and the combination treatment under pH controlled conditions. The pH buffered media (pH 6.0) indeed significantly prevented CLS reduction under the 2% glucose medium (**Figure 6C, E**). However, the longevity effects of methionine restriction or glutamic acid addition were not observed under buffered media under either normal or CR condition (**Figure 6C, D**). Nonetheless, the CLS extension effect of the combination treatment in buffered media was observed only under CR but not in normal glucose (**Figure 6C, D, E**). In sharp contrast to the un-buffered conditions, the pH buffered medium shortened the lifespan of the yeast cultured in 20-fold glutamic acid medium with 2% glucose (**Figure 6E**). Apparently, the lifespan extending capacity of CR was diminished by the buffered conditions (**Figure 6C, D**). Buffering was effective in increasing yeast longevity in the normal condition but not in CR condition as the media of former have lower pH values.

We then evaluated biomass production (**Figure 6F**, **S3E**), cell growth (**Figure S1**), pH (**Figure S5, S6**) and acetic acid of aging media (**Figure S5**). The combination treatment produced less biomass than that of glutamic acid addition, which had the highest biomass among the four treatments with or without pH buffering under 1E1N condition (**Figure 6F**) as well as under 1E5N condition (**Figure S3E**). We found that the combination treatment did not inhibit cell growth, while under 1E5N or 1E5N with methionine restriction slight delayed cell growth (**Figure S1**). Interestingly, addition of glutamic acid nulled this phenomenon, which suggests that high glutamic acid, not low methionine, might repair the amino acids imbalance induced by 1E5N.

For the pH values in buffered media, the four treatments did not cause apparent pH change under normal or CR condition as one would expect (**Figure S5A**). However, their lifespans were different (**Figure 6E**), indicating that nutrients composition is still an important factor to influence yeast CLS in the pH buffered media. In the un-buffered media, the combination treatment caused lower pH than that of high glutamic acid treatment in 1E1N and 1E5N media under normal condition (**Figure S6**). However, this does not mean that the additive longevity of glutamic acid and methionine is mainly due to the prevention of acidification of aging medium since the same phenomenon was observed in 1E1N media under CR condition without pH change (**Figure 6**, **S6**). Moreover, higher pH did not result in longer CLS among the four treatments in 1E5N media under normal and CR conditions (**Figure S3**, **S6**). Acetic acid production in aging media of the four treatments was showed to depend on nutrient composition and increased by the pH buffering (**Figure S5B, C**). The combination treatment did not cause substantially more acetic acid production compared to high glutamic acid intervention, while significant difference in CLS of both was observed. Taken together, these results strongly suggest that pH and acetic acid were not the major determinants of methionine and glutamic acid induced longevity.

### Conserved protein kinase Gcn2 mediates amino acids induced lifespan extension

**Figure 7:**
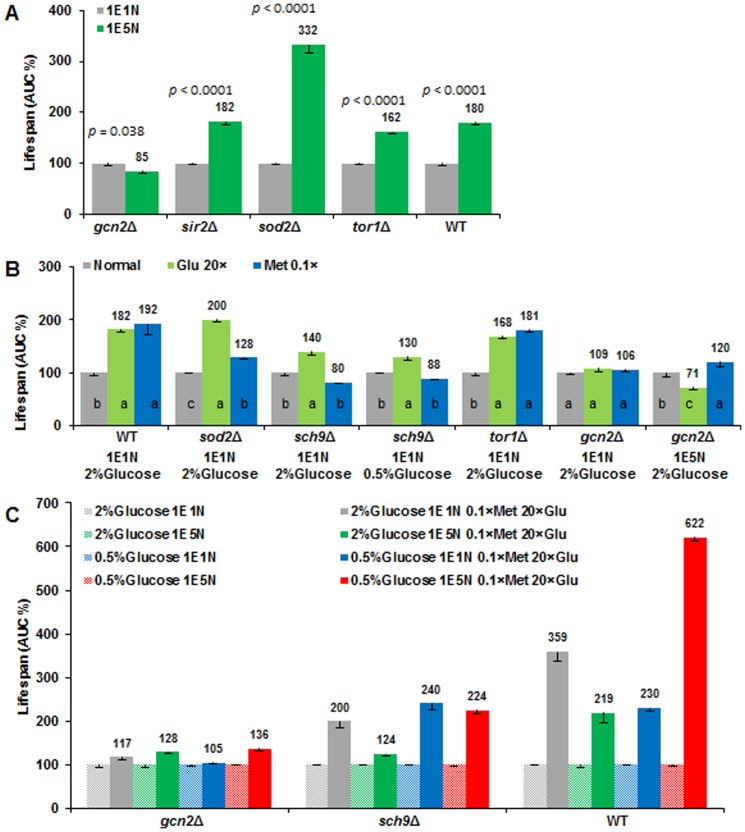
Conserved protein kinase Gcn2 mediates amino acid induced longevity. (**A**) Deletion of *GCN2* rather than other genes prevented 1E5N induced lifespan extension. AUC of each strain in the 1E1N medium was defined as 100%, respectively. Differences of the means of AUC% between 1E1N and 1E5N were determined by t-test. (**B**) Methionine and glutamic acid had distinct effects on lifespan regulation in *gcn2*Δ and *sch9*Δ. AUC of each strain in the control medium (Normal, or 1E1N) was defined as 100%, respectively. Differences of the means of AUC among normal, high glutamic acid (20× Glu) and low methionine (0.1× Met) were determined by Duncan’s multiple range test at *P*<0.01. (**C**) Deletion of *GCN2* was more effective than that of *SCH9* to impaired lifespan extension by the combination (0.1× Met + 20× Glu). AUC of each strain in the control (the four basic conditions: 1E1N or 1E5N with 0.5 or 2 % glucose) medium was defined as 100%, respectively. In most cases, significant difference was achieved between the combination and the control. All tests were based on SD media. 1E5N represents SD medium containing 1-fold EAA and 5-fold NEAA. Data are expressed as mean ± s.e.m. (n = 6). The survival curves are presented in **Figure S7**.

To explore the possible genetic mechanisms on how methionine, glutamic acid and glucose prompt longevity independently. We screened a number of genes that are evolutionarily conserved from yeast to human and compared their lifespan and survival curves in different media (**Figure 7** and **Figure S7**, respectively). Mitochondrial manganese superoxide dismutase (SOD2) was proposed as a downstream target of Tor/Sch9 nutrient signaling pathway for longevity extension by decreasing in part ROS levels in yeast, and deletion of *SOD2* has a shorter lifespan [18], [33], [34]. We observed that high NEAA (1E5N), low methionine, and high glutamic acid were able to extend lifespan of *sod2*Δ significantly (**Figure 7A, 7B**, **S7A, S7B**), which indicate the amino acid composition in SD medium plays an important role in regulation of lifespan in *sod2*Δ mutant.

Silent Information Regulator 2 (Sir2) has been proposed to mediate lifespan extension [35], [36]. Deletion of *SIR2* decreases RLS, whereas over-expression of *SIR2* increases RLS [37]. In contrast, deletion of *SIR2* was reported to extend CLS under control condition [19], [23], [24], [38]. We found that CLS of *sir2*Δ was extended by 182% in 1E5N medium in comparison with 1E1N SD medium (**Figure 7A**, **S7A**). This result suggested that Sir2 activity was not required for high NEAA induced CLS extension.

Reports have indicated that target of rapamycin (TOR) signaling may play a conserved role in mediating beneficial health and longevity effects associated with CR [14], [18], [39]. Deletion of *TOR1* increased lifespan significantly under normal condition but not under CR condition [20], [24], [40]. Here we showed that CLS of *tor1*Δ was extended by the NEAA, methionine restriction, or glutamic acid addition (**Figure 7A, 7B**, **S7A, S7B**). Altogether, we found that lifespan extension induced by high NEAA, methionine restriction, and glutamic acid addition could be independent of deletion of *SIR2*, *SOD2* and *TOR1* (**Figure 7**, **S7**).

Remarkably, Gcn2 was shown to impair lifespan extension induced by these amino acids treatment (**Figure 7A, B**). Gcn2 is one of major evolutionarily conserved protein kinases. It regulates amino acid homeostasis and protein synthesis through modulating amino acid biosynthesis in response to different amino acid deprivation in yeast [41]. Therefore, deletion of *GCN2* could result in cell function deficiency in modulating amino acids imbalance caused by methionine restriction, glutamic acid addition, or 1E5N. We found that the lifespan extending capacity of these treatment was impaired by the absence of *GCN2* (**Figure 7A, 7B**, **S7A, S7B**). The combination of methionine restriction and glutamic acid addition produced significant longevity in the *gcn2Δ* strain under four different media compositions (**Figure 7C**). A similar trend is observed for the *sch9Δ* strain (**Figure 7C**). This indicated that Gcn2 might be a major target in regulating amino acid metabolism, which, in turn, influences yeast chronological aging.

It is well known that the longevity of *sch9*Δ strain is CR-dependent. For example, deletion of *SCH9* extended lifespan in normal condition but not under CR condition in comparison with that of WT strain (**Figure S7B**). Sch9 was proposed as a highly conserved nutrient-sensing factor to regulate aging, cell growth, cell size, and stress resistance through controlling protein synthesis [40], [42], [43]. However, methionine restriction could not extend CLS of *sch9*Δ strain under normal and CR conditions, which means methionine induced longevity required Sch9 activity. On the other hand, glutamic acid extended CLS in *sch9*Δ mutant but not in *gcn2*Δ mutant (**Figure 7B**, **S7B**). Altogether, the longevity via modification of amino acids required, in part, Gcn2 activity, while Sch9 was necessary for methionine and glucose restriction induced longevity. Thus, the distinct mechanisms could somewhat explain the additive longevity effect of methionine restriction, glutamic acid addition and CR although further verification is needed to support the mechanisms.

The pH values of *sch9Δ* at day 2 fell within a very narrow range of 3.4 to 4.0 for 2% glucose and 4.3 to 5.5 for 0.5% glucose (**Figure S6**). Hence it is apparent that the pH values of the media do not correlate with the CLS of the yeast. Similarly, the pH values of the media of *gcn2Δ* do not correlate with the CLS (**Figure S6**). We also found that acetic acid was not a key determinant of CLS in *sch9*Δ and *gcn2*Δ, since higher acetic acid had longer lifespan in some cases (**Figure S8**). It is well known that CR shortens lifespan of *sch9*Δ, which was also observed in this study (**Figure S7B**). However, CR resulted in less acetic acid production (**Figure S8**). Thus these data might imply that deletion of *SCH9* and *GCN2* mediate CLS independently of acetic acid and acidification of aging medium [15].

## Discussion

In this study, we used a high throughput screening assay to comprehensively evaluate the CLS-extending capacity of amino acids. We found the ratio of NEAA and EAA caused great changes in CLS, biomass production, cell growth, pH and acetic acid of aging medium. Increase or decrease of an individual amino acid had little effect on CLS change in most cases except for methionine restriction and glutamic acid addition, which substantially resulted in CLS extension. Furthermore, our data showed that the two NEAAs could CR-independently extend yeast CLS through distinct mechanisms targeting evolutionarily conserved protein kinase, such as Sch9 and Gcn2.

Methionine has been reported to play a critical role in regulation of lifespan in rat, fly and yeast [6], [10], [12], [44], [45], [46]. Methionine restriction (by 80%) increased median and maximum lifespan of rats by 30% and 40%, respectively [13]. Later studies indicated that methionine restriction delayed the onset of age-dependent pathologies and extended lifespan through control of adiposity and insulin resistance in rats and mice independently of CR [12], [45]. Although the mechanisms of methionine restriction induced lifespan extension was not fully understood, a few studies suggested that it was different to CR at the molecular level in mammalian. For example, CR increased the phosphorylation of ERK, JNK2, p38, mTOR and 4EBP1, while no such effect was observed from methionine restriction [47].

In *Drosophila*, methionine restriction (by 67%) extended maximum and mean lifespan by 2.4% and 10.5%, respectively. Severe restriction (by 88%) did not further extend maximum and mean lifespan [48]. In *S. cerevisiae*, a study reported that reduction in methionine (0.1×) increased average replicative lifespan (RLS), and high methionine (10×) slightly shortened RLS [44]. Altogether, these results were in agreement with our observation that methionine restriction caused CLS extension but high level of methionine shortened CLS (**Figure 5A-D**).

It should be noted that intake of high amount of methionine is very toxic to both young and adult mammals, and this toxicity far exceeds that produced by the excess intake of any other amino acid [49]. In addition, our study suggested that methionine restriction may share some of the effects with CR, i.e. either methionine restriction or CR caused lifespan reduction in *sch9*Δ strain (**Figure S7B**), which indicates they induce longevity via diminishing *Sch9* activity. Methionine restriction induced CLS extension seemed to require *Sch9* and *Gcn2* activity (**Figure 7B**). Overall, these data partially supported the notion that methionine was able to mediate some of evolutionarily conserved longevity signaling pathways to induce lifespan extension among different species.

Glutamic acid (or glutamate under human physiological pH) is the most abundant free amino acid in brain as a key excitatory neurotransmitter of central nervous system and is primarily linked to the pathogenesis of many neurological diseases or disorders, such as Alzheimer’s disease, amyotrophic lateral sclerosis, autism, cerebral ischemia, depression, epilepsy, Huntington disease, multiple sclerosis, Parkinson’s disease, schizophrenia and traumatic brain injury [50], [51]. In *S. cerevisiae*, glutamate plays fundamental roles in amino acid metabolism, tricarboxylic acid (TCA) cycle, and glutathione synthesis. Glutamate can be degraded by a NADP^+^-dependent glutamate dehydrogenase (GLDH) encoded by *GDH2* to α-ketoglutarate and ammonia. Glutamate can also be biosynthesized from α-ketoglutarate and ammonia by two NAD^+^-dependent GLDH Gdh1 and Gdh3 [52]. Gdh1 was proposed to be more suitable for regulation of glutamate production during exponential phase, while Gdh3 might be more important to mediate glutathione biosynthesis for resistance to stress-induced apoptosis and chronological aging during stationary phase [53].

In both yeast and mammalian cells, glutathione is a crucial metabolite for stress resistance and its biosynthesis requires glutamate. It was also shown that glutamate could suppress reactive oxygen species (ROS) accumulation to prevent thermal and oxidative stress-induced apoptosis in the stationary cells of *GDH3* deletion strain [53]. In this study, it is the first to report the lifespan-extending activity of glutamic acid. We found that glutamic acid caused wild-type yeast CLS extension in a dose-response manner (**Figure 5E-H**), as well as protected against the fast loss of viability during chronological aging in *SOD2*-null strain (**Figure 7B**). Yeast has two SOD genes, cytoplasmic copper-zinc superoxide dismutase (*SOD1*) and mitochondrial manganese superoxide dismutase (*SOD2*). The lack of either of the two SODs causes attenuation in replicative and chronological aging due to high oxidative damage induced by ROS in the cell [54], [55]. Hence, glutamic acid extends CLS probably via enhancing ROS stress resistance of yeast cells. In addition, high glutamic acid appears to significantly increase biomass under conditions in which it extends CLS (**Figure 5** **,** **6**, **S3**). This suggests that high glutamic acid plays a role in biomass synthesis that may be important during extension of CLS. Our findings justify further investigations of lifespan extending activity of glutamic acid in other animal models or in-depth studies of the longevity mechanisms in yeast.

Recent studies suggested that the low glucose concentration prompted yeast lifespan extension was primarily due to decreased production of acetic acid and reduced acidification of medium for two possible reasons: (1) acetic acid was identified as an extracellular mediator of cell death during chronological aging. It was demonstrated that environmental treatment to reduce or eliminate acetic acid increased CLS. These treatments include applying CR, using non-fermentable carbon source, or transferring cells to water [29]; (2) pH buffering was demonstrated to protect against reduction in RLS and CLS in yeast [30], [32], [56]. Extracellular acidification of the culture medium could cause intracellular damage that subsequently limited the cell replicative potential. The reduced RLS and CLS could be restored by buffering the pH of medium to 6.0 [30].

It is thus reasonable to suggest that the amino acid induced CLS alterations are probably due to the changes in acetic acid and pH of aging media. In this study, we measured acetic acid production and pH of stationary phase media. The data showed that ratio of EAA/NEAA, methionine and glutamic acid extended CLS was not always accompanied with lower acetic acid concentration and higher pH of the medium. Moreover, CLS of wild-type, *sch9*Δ and *gcn2*Δ also had poor correlation with the acetic acid production and pH values of the media. The fact that amino acid treatment caused significant CLS changes in pH buffered media further indicated that the pH might not be a primary factor to limit CLS.

Glutamic acid addition coupled with methionine and glucose restriction resulted in an optimal medium for yeast lifespan extension, which could not be further enhanced by buffering the pH of the media, modifying EAA and NEAA composition, or deleting the longevity gene *SCH9*. Methionine and glucose restriction were also reported to cause lifespan extension in other organisms, including mammals [12], [13]. Furthermore, our data suggested the three treatments individually functioned on highly evolutionarily conserved kinases, such as Sch9 and Gcn2, which have been implicated in nutrients metabolism, cell development, stress resistance and aging [40], [41]. Thus, our results imply that the extraordinary yeast lifespan extension capacity of the combination of glutamic acid addition, methionine and glucose restriction might also be realized in mammals.

## Supporting Information

Figure S1 **Glutamic acid addition, methionine and glucose restriction do not inhibit yeast growth.** Wild-type (BY4742) yeast were cultured in 1E1N (normal) and 1E5N media without (control) or with low methionine (0.1× Met, 8 mg/L), high glutamic acid (20× Glu, 2 g/L) or a combination of both (0.1× Met + 20× Glu) under un-buffered normal (2% glucose, **A**) and CR (0.5% glucose, **B**) conditions or under pH (6.0) buffered normal glucose (**C**) and CR (0.5% glucose, **D**) conditions. Yeast (≈ 1×10^4^ cells) cells were grown in each well of 96-well microplate containing 100 µL of different media. The cell population was monitored with a microplate reader by recording the OD every 5 min at 660 nm. The pH neutralization was prepared by buffering the pH of medium using citrate phosphate buffer solution (64.2 mM Na_2_HPO_4_ and 17.9 mM citric acid, pH 6.0). All tests were based on SD media. 1E5N represents SD medium containing 1-fold EAA and 5-fold NEAA.
(TIF)

Figure S2 **Comparison of pH and acetic acid of aging media containing various levels of methionine and glutamic acid.** The pH of aging media (day 2) had only little change among various media. Differences in means of the pH values of different aging media were analyzed by Duncan’s multiple range test at *P*<0.01. Acetic acid was increased by adding methionine and reducing glutamic acid in media. Data are expressed as mean ± s.d.(n = 3) and datum 0.0 means not detectable.
(TIF)

Figure S3 **Glutamic acid and methionine have no additive effect on longevity of yeast cultured in the medium supplying with 5-fold NEAA.** Survival curves of wild-type yeast were cultured in un-buffered normal (2% glucose, **A**) and CR (0.5% glucose, **B**) conditions or pH (6.0) buffered normal glucose (**C**) condition without (1E5N, control) or with low methionine (0.1× Met), high glutamic acid (20× Glu) or a combination of both (0.1× Met + 20× Glu). CLS (**D**) and biomass (**E**) comparison of wild type yeast were cultured in different media. AUC represents the survival integral for lifespan comparison. AUC of the control (1E5N with 2% glucose) was defined as 100%. Biomass of SD medium (1E5N with 2% glucose) was defined as 100%. Data are expressed as mean ± s.d. (n = 6) and compared using Duncan’s multiple range test at *P*<0.05. Different lowercase letters in columns indicate significant difference.
(TIF)

Figure S4 **The combination treatment is sufficient to extend yeast lifespan.** Survival curves of wild type yeast (BY4742) were cultured in different media. The combination treatment (0.1× Met + 20× Glu) greatly extends lifespan in CR, 1E5N and pH buffered conditions, except in buffered 1E1N medium with 2% glucose. All tests are based on SD media. 1E5N represents SD medium containing 1-fold EAA and 5-fold NEAA.
(TIF)

Figure S5 **Acetic acid may be not the cause of lifespan shortening in buffered media (pH 6.0).** (**A**) The pH of the four buffered media changed slightly under normal and CR conditions. The pH was measured at stationary phase (day 2) of yeast cultured in 1E1N medium without (normal) or with low methionine (0.1× Met), high glutamic acid (20× Glu) or a combination of both (0.1× Met + 20× Glu). Acetic acid accumulation in the four aging media (control, 0.1× Met, 20× Glu and 0.1× Met + 20× Glu) under normal and CR conditions with (**B**) or without pH buffering (**C**). Data expressed as mean ± s.d (n = 3) and datum 0.0 means not detectable.
(TIF)

Figure S6 **Comparative evaluation of the pH of aging media in WT,** ***sch9*** **Δ and** ***gcn2*** **Δ.** The pH was measured at stationary phase (day 2) of yeast cultured in 1E1N (normal) and 1E5N media without (control) or with low methionine (0.1× Met), high glutamic acid (20× Glu) or a combination of both (0.1× Met + 20× Glu). 0.5% glucose (CR) caused higher pH than 2% glucose in the eight media in the three strains, while the pH varied slightly among the four treatments (control, 0.1× Met, 20× Glu and 0.1× Met + 20× Glu) under the same condition (1E1N or 1E5N) and the same strain. Differences in means of the pH values of different media under normal (2% glucose) or CR conditions, respectively, were analyzed by Duncan’s multiple range test at *P*<0.01 and different lowercase letters in columns indicate significant difference. Data expressed as mean ± s.d (n = 3).
(TIF)

Figure S7 **Comparative evaluation of the amino acid induced longevity in different single gene deletion strains.** (**A**) Survival curves of *gcn2*Δ, *sir2*Δ, *sod2*Δ, and *tor1*Δ were cultured in 1E1N or 1E5N with 2% glucose medium. (**B**) Survival curves of *gcn2*Δ, *sch9*Δ, *sod2*Δ, and *tor1*Δ were grown in different media with high glutamic acid (20× Glu) or low methionine (0.1× Met). (**C**) Survival curves of *gcn2*Δ, and *sch9*Δ were grown in different media with/without the combination of glutamic acid and methionine (0.1× Met + 20× Glu). Survival curve are the mean of six parallel measurements. The statistical analysis of significant difference for the CLS comparison is presented in **Figure 7**.
(TIF)

Figure S8 **The acetic acid production of aging media in** ***sch9*** **Δ and** ***gcn2*** **Δ.** 0.5% glucose (CR) caused lower acetic acid accumulation in aging media than 2% glucose in most cases. The acetic acid was measured at stationary phase (day 2) of yeast cultured in 1E1N (Normal) and 1E5N media without (control) or with low methionine (0.1× Met), high glutamic acid (20× Glu) or a combination of both (0.1× Met + 20× Glu). Data are expressed as mean ± s.d. (n = 3).
(TIF)
