# Supplementary figures and images for: Independent and Additive Effects of Glutamic Acid and Methionine on Yeast Longevity
Source: PLoS One. 2013 Nov 7;8(11):e79319. doi: 10.1371/journal.pone.0079319 (PMC3820698; doi:10.1371/journal.pone.0079319)

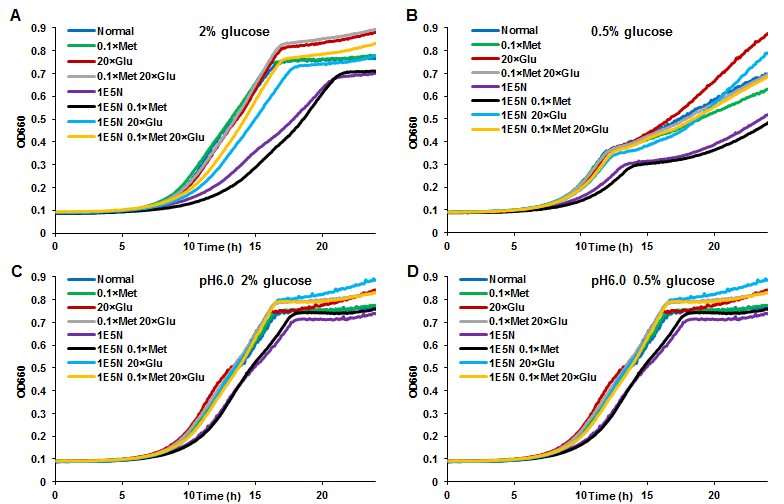

Supplement: Figure S1 — Glutamic acid addition, methionine and glucose restriction do not inhibit yeast growth. Wild-type (BY4742) yeast were cultured in 1E1N (normal) and 1E5N media without (control) or with low methionine (0.1× Met, 8 mg/L), high glutamic acid (20× Glu, 2 g/L) or a combination of both (0.1× Met + 20× Glu) under un-buffered normal (2% glucose, A) and CR (0.5% glucose, B) conditions or under pH (6.0) buffered normal glucose (C) and CR (0.5% glucose, D) conditions. Yeast (≈ 1×10^4^ cells) cells were grown in each well of 96-well microplate containing 100 µL of different media. The cell population was monitored with a microplate reader by recording the OD every 5 min at 660 nm. The pH neutralization was prepared by buffering the pH of medium using citrate phosphate buffer solution (64.2 mM Na2HPO4 and 17.9 mM citric acid, pH 6.0). All tests were based on SD media. 1E5N represents SD medium containing 1-fold EAA and 5-fold NEAA. (TIF) [file pone.0079319.s001.tif]

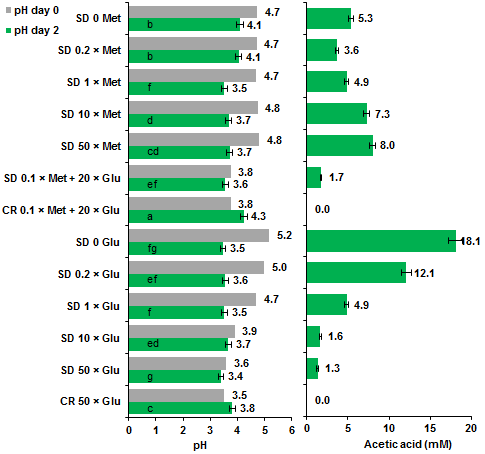

Supplement: Figure S2 — Comparison of pH and acetic acid of aging media containing various levels of methionine and glutamic acid. The pH of aging media (day 2) had only little change among various media. Differences in means of the pH values of different aging media were analyzed by Duncan’s multiple range test at P<0.01. Acetic acid was increased by adding methionine and reducing glutamic acid in media. Data are expressed as mean ± s.d.(n = 3) and datum 0.0 means not detectable. (TIF) [file pone.0079319.s002.tif]

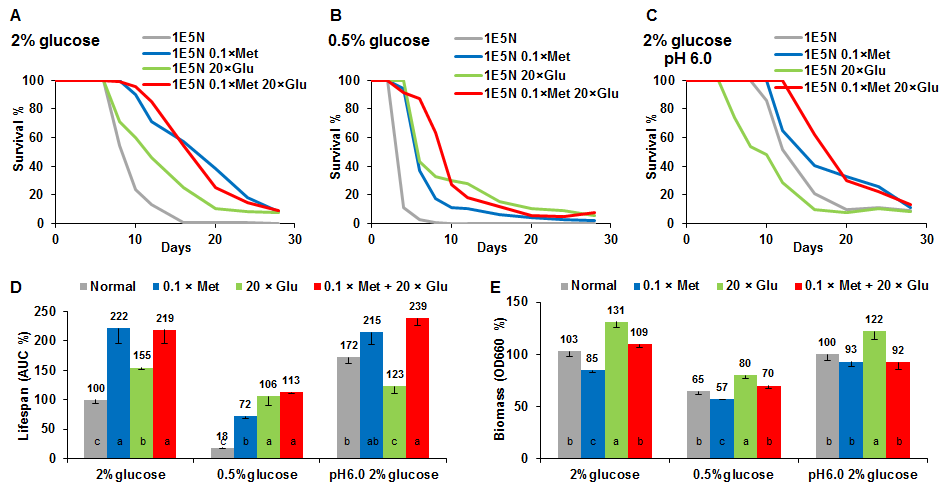

Supplement: Figure S3 — Glutamic acid and methionine have no additive effect on longevity of yeast cultured in the medium supplying with 5-fold NEAA. Survival curves of wild-type yeast were cultured in un-buffered normal (2% glucose, A) and CR (0.5% glucose, B) conditions or pH (6.0) buffered normal glucose (C) condition without (1E5N, control) or with low methionine (0.1× Met), high glutamic acid (20× Glu) or a combination of both (0.1× Met + 20× Glu). CLS (D) and biomass (E) comparison of wild type yeast were cultured in different media. AUC represents the survival integral for lifespan comparison. AUC of the control (1E5N with 2% glucose) was defined as 100%. Biomass of SD medium (1E5N with 2% glucose) was defined as 100%. Data are expressed as mean ± s.d. (n = 6) and compared using Duncan’s multiple range test at P<0.05. Different lowercase letters in columns indicate significant difference. (TIF) [file pone.0079319.s003.tif]

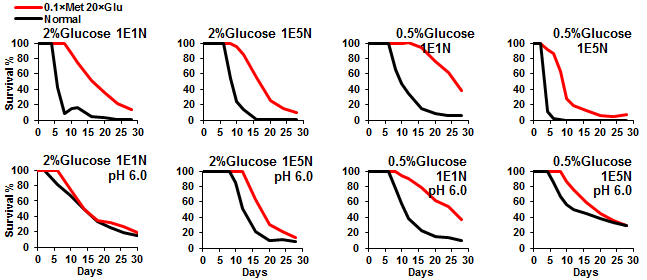

Supplement: Figure S4 — The combination treatment is sufficient to extend yeast lifespan. Survival curves of wild type yeast (BY4742) were cultured in different media. The combination treatment (0.1× Met + 20× Glu) greatly extends lifespan in CR, 1E5N and pH buffered conditions, except in buffered 1E1N medium with 2% glucose. All tests are based on SD media. 1E5N represents SD medium containing 1-fold EAA and 5-fold NEAA. (TIF) [file pone.0079319.s004.tif]

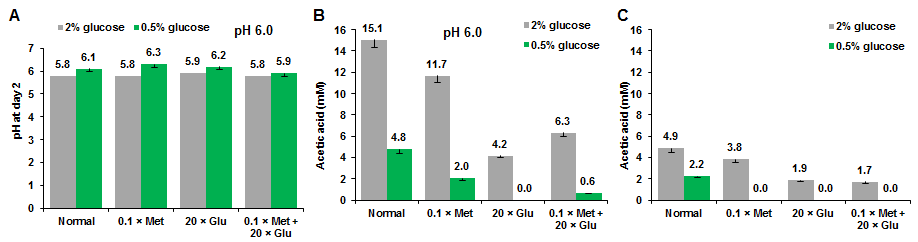

Supplement: Figure S5 — Acetic acid may be not the cause of lifespan shortening in buffered media (pH 6.0). (A) The pH of the four buffered media changed slightly under normal and CR conditions. The pH was measured at stationary phase (day 2) of yeast cultured in 1E1N medium without (normal) or with low methionine (0.1× Met), high glutamic acid (20× Glu) or a combination of both (0.1× Met + 20× Glu). Acetic acid accumulation in the four aging media (control, 0.1× Met, 20× Glu and 0.1× Met + 20× Glu) under normal and CR conditions with (B) or without pH buffering (C). Data expressed as mean ± s.d (n = 3) and datum 0.0 means not detectable. (TIF) [file pone.0079319.s005.tif]

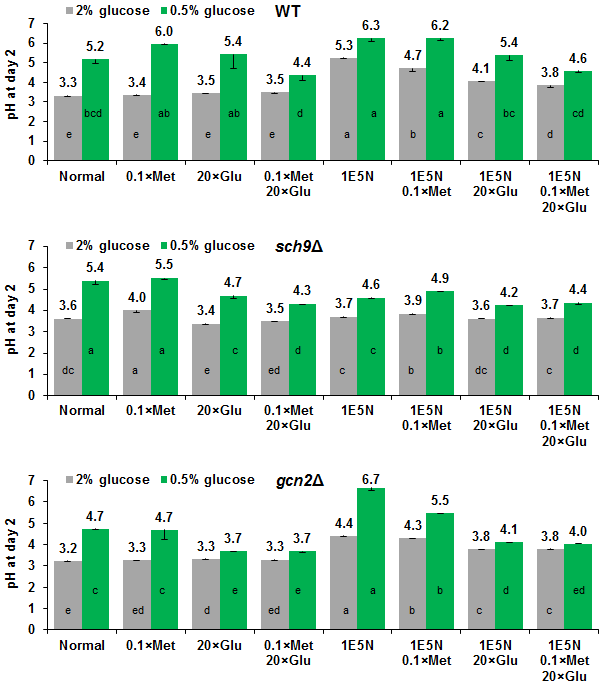

Supplement: Figure S6 — Comparative evaluation of the pH of aging media in WT, sch9 Δ and gcn2 Δ. The pH was measured at stationary phase (day 2) of yeast cultured in 1E1N (normal) and 1E5N media without (control) or with low methionine (0.1× Met), high glutamic acid (20× Glu) or a combination of both (0.1× Met + 20× Glu). 0.5% glucose (CR) caused higher pH than 2% glucose in the eight media in the three strains, while the pH varied slightly among the four treatments (control, 0.1× Met, 20× Glu and 0.1× Met + 20× Glu) under the same condition (1E1N or 1E5N) and the same strain. Differences in means of the pH values of different media under normal (2% glucose) or CR conditions, respectively, were analyzed by Duncan’s multiple range test at P<0.01 and different lowercase letters in columns indicate significant difference. Data expressed as mean ± s.d (n = 3). (TIF) [file pone.0079319.s006.tif]

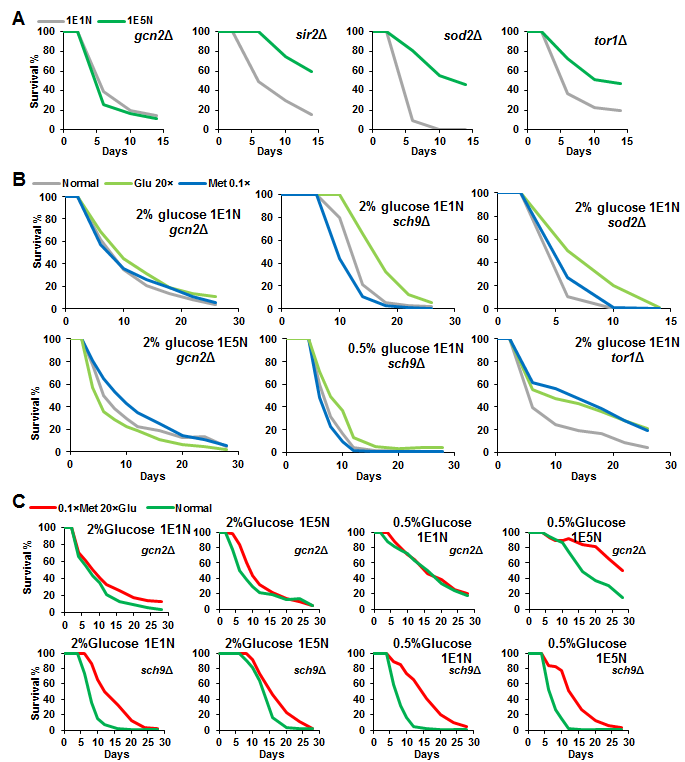

Supplement: Figure S7 — Comparative evaluation of the amino acid induced longevity in different single gene deletion strains. (A) Survival curves of gcn2Δ, sir2Δ, sod2Δ, and tor1Δ were cultured in 1E1N or 1E5N with 2% glucose medium. (B) Survival curves of gcn2Δ, sch9Δ, sod2Δ, and tor1Δ were grown in different media with high glutamic acid (20× Glu) or low methionine (0.1× Met). (C) Survival curves of gcn2Δ, and sch9Δ were grown in different media with/without the combination of glutamic acid and methionine (0.1× Met + 20× Glu). Survival curve are the mean of six parallel measurements. The statistical analysis of significant difference for the CLS comparison is presented in Figure 7 . (TIF) [file pone.0079319.s007.tif]

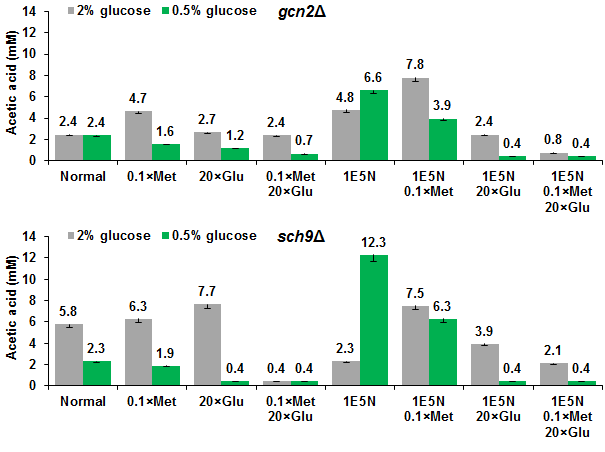

Supplement: Figure S8 — The acetic acid production of aging media in sch9 Δ and gcn2 Δ. 0.5% glucose (CR) caused lower acetic acid accumulation in aging media than 2% glucose in most cases. The acetic acid was measured at stationary phase (day 2) of yeast cultured in 1E1N (Normal) and 1E5N media without (control) or with low methionine (0.1× Met), high glutamic acid (20× Glu) or a combination of both (0.1× Met + 20× Glu). Data are expressed as mean ± s.d. (n = 3). (TIF) [file pone.0079319.s008.tif]
